# diArk – a resource for eukaryotic genome research

**DOI:** 10.1186/1471-2164-8-103

**Published:** 2007-04-17

**Authors:** Florian Odronitz, Marcel Hellkamp, Martin Kollmar

**Affiliations:** 1 Department of NMR-based Structural Biology, Max-Planck-Institute for Biophysical Chemistry, Am Fassberg 11, 37077 Goettingen, Germany

## Abstract

**Background:**

The number of completed eukaryotic genome sequences and cDNA projects has increased exponentially in the past few years although most of them have not been published yet. In addition, many microarray analyses yielded thousands of sequenced EST and cDNA clones. For the researcher interested in single gene analyses (from a phylogenetic, a structural biology or other perspective) it is therefore important to have up-to-date knowledge about the various resources providing primary data.

**Description:**

The database is built around 3 central tables: species, sequencing projects and publications. The species table contains commonly and alternatively used scientific names, common names and the complete taxonomic information. For projects the sequence type and links to species project web-sites and species homepages are stored. All publications are linked to projects. The web-interface provides comprehensive search modules with detailed options and three different views of the selected data. We have especially focused on developing an elaborate taxonomic tree search tool that allows the user to instantaneously identify e.g. the closest relative to the organism of interest.

**Conclusion:**

We have developed a database, called diArk, to store, organize, and present the most relevant information about completed genome projects and EST/cDNA data from eukaryotes. Currently, diArk provides information about 415 eukaryotes, 823 sequencing projects, and 248 publications.

## Background

Since the publication of the first complete genome sequence of an eukaryote, *Saccharomyces cerevisiae *[1], the genome sequencing community has produced highly advanced drafts of many other eukaryotes. The past few years have thus seen the rise of a completely new field in biology that is described as comparative genomics [2]. Initial results have shown that whole genome comparisons are important to improve the annotation of genes and transcripts of a genome. It has also been demonstrated that not only genome sequences of organisms spread over all kingdoms of eukaryotic life are needed but also many of closely related organisms [3]. These results have lead to the Fungi genome initiative representing the widest sampling of genomes from any eukaryotic kingdom, the mammalian genome project aimed to expand the genome coverage of mammals, and the *Drosophila *species sequencing project intended to establish methods for comparative genomics among other things. Thus, it is evident that future sequencing efforts have to include both further taxonomic sampling and closely related organisms.

In many research areas it is important to have access to DNA data and DNA samples of as many organisms as possible. For example, in structural biology there is a strong tendency to also work with homologs of other organisms to enhance the chance of obtaining structural data because cloning and protein expression are not as time consuming as they were some years ago [4]. Reconstructing phylogenetic relationships between species or proteins is another expanding topic and it is clear that the addition of further sequence data improves the significance of the analyses by enhancing the statistics and therefore limiting the negative effects of outliers [5].

Two main databases provide access to lists of completed and ongoing eukaryotic genome projects. The Genomes OnLine Database (GOLD [6]) presents information on sequencing projects sorted according to the three major lineages of the tree of life. In addition, GOLD distinguishes between published and ongoing projects but lists some of the completed and not yet published genomes with the published projects. GOLD also contains some limited information about genome sizes, GC contents, and contact persons. The International Sequencing Consortium (ISC [7]) has established a web-site to provide up-to-date information about eukaryotic genome sequencing projects of member institutions. The list also provides information about the sequencing product, the strategy applied and a proposed timetable. Both databases list all funding agencies, the sequencing centers, and very basic taxonomic information about all species. However, the taxonomic information is that limited that the user cannot identify for example the closest homolog to his organism of interest. In addition, only a very limited amount of alternative scientific names and no common names are provided, and there is also only a limited number of links to access the primary data.

Here, we present the web-interface to diArk (digital ark) providing information on eukaryotic sequencing projects that resulted either in at least preliminary assemblies of genome data or a substantial amount of EST or cDNA data. In the center of the database are extensive species-related information (commonly and alternatively used scientific names, common names, and complete taxonomies) and much information about the respective species sequencing projects. Apart from the up-to-date status of the data our focus has been on a feature rich user interface with comprehensive and easy-to-use search capabilities.

## Construction and Content

### Technologies

The system is running on UNIX (OS X and Linux) systems. The database management system is PostgreSQL [8]. As web application framework we chose Ruby on Rails [9] since it has the advantage of rapid and agile development while keeping the code well organized. Part of this framework is an implementation of Active Record [10] which is an O/RM (Object-relational Mapping) system making database integration into an object oriented program considerably easier.

The web pages are generated as XML. The site makes extensive use of Ajax (Asynchronous JavaScript and XML) in order to present the user with a feature rich interface while minimizing the amount of transferred data. All technologies used are freely available and open source.

### Database

The unique requirements of the system demand a custom database schema (Additional file 1). At the center of the database are three interconnected tables: species, projects and publications (see additional file 1: Database schema). The species table holds all information about the different scientific and common names, so that every species can be found even when the user does not know the exact scientific name. A comment field may contain general information about the corresponding species, the specific strain used, or common and divergent features compared to closely related organisms. Each species record is linked to a tree-like data-structure representing its taxonomy. Through this hierarchical tree, it is possible to easily select sets of species in the same taxon. The maintenance of the taxonomy tree is an automated procedure, which is triggered by the database upon insertion of new species. A delegation server receives messages from the database and starts a script to update the taxonomy tree.

The projects table contains details concerning a specific sequencing effort, such as its type (genomic DNA or EST/cDNA) and a link to the web-page providing the primary data. The term completeness is intended to describe the coverage of the genome. In this respect, EST/cDNA data is always incomplete as most genes are either only partially or not at all covered. Genomic sequencing is thought to be complete if a certain quality and coverage of the assembly is reached. Genome sequences with low assembly coverages (<3×) and/or short assembled contigs (a few kbp) do not provide enough information to reconstitute even medium sized genes and are also considered incomplete (e.g. the mammalian 2 × coverage sequencing projects). Each project may be assigned to a reference, a term we use for the large-scale sequencing centers (e.g. the DOE Joint Genome Institute) or community species homepages (e.g. FlyBase). However, for many species, the sequence information is not available via a dedicated species home page but only via GenBank. Therefore the "GenBank" links provide BLAST search forms including the corresponding database (some data is only available from the WGS, other from the EST database) and the corresponding species name. The projects table is always linked to a species and, in case they exist, to one or more publications.

The publications table stores all relevant information about a publication like author, title, year and journal. We included publications that refer to specific cDNA datasets (e.g. the large scale cDNA sequencing of the nematodes), or that refer to the first description of the genome sequence (e.g. the publication of the *Osterococcus tauri *genome). These interconnected sets of species, projects and publications form the base of the search function. For example, searching for a species also returns projects and publications. Data entry is done using the iiwi system (Odronitz F., Lampetsdoerfer T., Dietrich D., unpublished results [12]) allowing for remote editing and access control.

## Utility and Discussion

Hundreds of sequencing projects have been started in the past few years and thus the number of projects offering access to first assemblies is increasing rapidly. However, a database providing access to the primary data (genomic DNA or cDNA/EST data) of all sequenced organisms does not exist. For example, the DOE Joint Genome Institute provides access to 23 completely sequenced eukaryotes via dedicated species project pages and the data for another 3 eukaryotes via ftp server. However, the assembly data of only 9 species have already submitted to NCBI, although the data of another one has already been published. At NCBI, there are two possibilities to BLAST against genomic assembly data: directly using e.g. TBLASTN choosing the WGS database or by selecting one of the genomicBLAST tables. However, the supposedly complete table of eukaryotic genomes does not include the plant genomes. There are also strong discrepancies between the WGS database and the assemblies available via genomicBLAST. The WGS database contains 145 species while the genomicBLAST tables list only 130 organisms of which 2 are redundant. Missing species in the genomicBLAST tables comprise for example the fish *Gasterosteus aculeatus*, the plants *Ricinus communis *and *Populus trichocarpa*, and the fungus *Batrachochytrium dendrobatidis*. Even more complicating, both databases often provide different assembly versions of the genomes (e.g. v3 of the *Apis mellifera *genome in the WGS database and v4.1 via the genomicBLAST tables). These numbers show that there is a strong need for a universal database providing access to all the different sequencing projects.

diArk has been developed to store, organize and present information about sequencing projects, that have either produced preliminary or final assemblies of genome data, or that have resulted in substantial amounts of EST or cDNA data. The aim was to provide the best overview possible about the different projects so that researchers get easy access to the primary data to increase for example the taxon sampling in their phylogenetic analyses. Altogether, diArk provides links to 209 genome assemblies and to the EST/cDNA data of 291 species (as of 12-Dec-2006). diArk does not include species for which only sequence reads are available. Given the already existing amount of completed genomes and the accumulated know-how in the sequencing centers it would not be reasonable for single researchers to build their own assemblies. We decided to not include those species until at least a draft assembly is available. Next to be up-to-date and complete, the most important requirement for diArk is a powerful and easy to use search tool.

### Web Interface

Great attention has been paid to a versatile yet easy to use web interface. We think that accessibility and high quality representation is key to a productive usage of the system. diArk encompasses a live web front end that is generated from the content of the database at each request and thus always reflects the current data. The database is searched using modules that can be combined in chains. There are five different modules each providing specific options: a module for the full-text search in all species names, a taxonomy search module, a module to select specific groups of species, a module to search sequencing project related data, and a publication search module. A search can consist of any combination of modules and their options. By adding further search modules the user can successively refine the search and narrow down the result list. For each module the resulting selection of species, projects and publications is shown, providing additional context. If a new module is added the options available will be restricted by the selection from the previous modules. At any time, the search options for every module can be changed and modifications are propagated down the chain reapplying previous user actions.

Species can be searched for in two ways. The full-text search module provides an autocompletion input field to search the list of scientific and common species names. The taxonomy search module offers tables containing specific subsets like a selection of major taxa or a range of model organisms. In addition, this module provides a taxonomic tree representation for the selection of taxa and species. Taxa and species can be browsed and selected by expanding/collapsing and including/excluding subsections of the tree, or by using shortcuts or auto-completion fields. If the dataset has been restricted by previous modules (e.g. the selection of a specific reference), excluded species and taxa are disabled in the tables.

All searches can be saved and re-run. The searches are saved purely as instructions on how to search the database. This means that if the underlying data has changed since the last run, the options set by the user will be reapplied to the data, possibly resulting in a different set of results. Based on this mechanism, we implemented an alert service that is running saved searches on a regular basis and alerts the user by email as soon as the results have changed. This enables highly customized searches to be re-run automatically in order to monitor a specific subset of the data.

### Web Services

In order to make our data available programmatically to other researchers we implemented a web service that supports XML-RPC and SOAP. The methods allow a remote program to retrieve the full data on species, publications and projects as well as the relations between different types of records. Additionally we offer a method that is equivalent to the auto-completion of the interface: When a string is given as an argument, the web service returns an array of species-IDs where the string occurs in any one of the name fields. We also make available a range of methods related to taxonomy: Taxonomy records (currently 1906), their respective children and parent as well as all species within a taxon can be retrieved. For any given species an array of taxonomy records representing their ancestry is available.

With these mechanisms we enable other programmers to conveniently construct complex queries on diArk's interconnected data without knowing about the internals.

### Case Study

**Figure 1:**
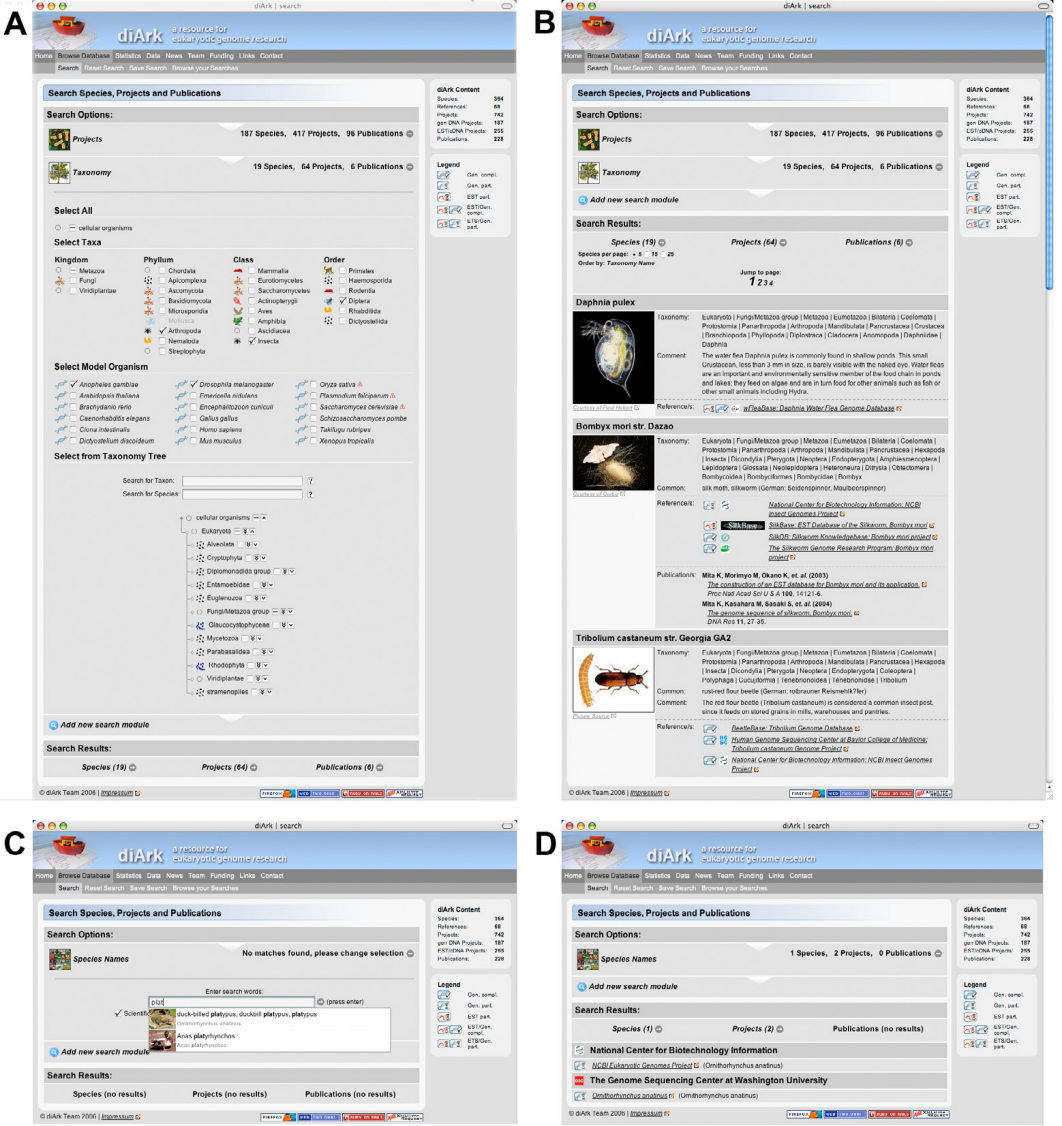
**Screenshots of diArk's web-interface**. The screenshots highlight parts of the searches described in the case study.

Alice wants to see which Arthropoda genomes have already been sequenced (Figure 1). In the taxonomic search module all species are listed regardless whether only cDNA or genomic DNA data is available. Therefore, she would have to first select "genomic DNA data" in the projects module. Afterwards Alice could either browse through the taxonomic tree to the Arthropoda and the underlying species or select the Arthropoda from the tax table (Figure 1A) and view all contents in the species result view (Figure 1B).

Bob wants to know whether platypus has already been sequenced, and if a genome assembly exists, to see the list of web-sites to get access to the genome data. By typing "plat" into the species autocompletion form of the species names search module (or the taxonomy search module) he finds that the scientific name of platypus is Ornithorhynchus anatinus and that there is another hit with Anas platyrhynchos (Figure 1C). Having selected Ornithorhynchus anatinus Bob may either choose to view the complete information connected to this organism by choosing the species view, or to view only the list of links to sequencing projects in the project view (Figure 1D).

### Related Work

There is only one other serious compilation of genome sequencing projects, the GOLD database [6]. GOLD comprises data of all three major lineages of life, the bacteria, the archaea and the eukaryotes. GOLD lists 674 eukaryotic sequencing projects (genome and cDNA sequencing) of which 44 are marked as published and another 13 as completed of which 4 are not publicly available. In comparison, we have found 209 genome projects (161 completed, 62 published) and included them in diArk. The major focus of GOLD seems to list all funded and ongoing sequencing projects so that researchers and sequencing consortia get an overview and help in the decision about new target species. Therefore, GOLD includes a very thorough compilation of the corresponding species sequencing centers, the funding agencies, and contact persons. On the other hand, the taxonomic information in GOLD is very limited, only a few alternative scientific names are listed and no common names are provided. In addition, only a limited number of direct links to the assembly data are given. Another major drawback of GOLD is being incomplete and not up-to-date. For example, 15 % of the links associated with eukaryotic sequencing projects do not work (397 of 2644 total). In addition, many projects are still listed as "incomplete" although assembly data became available years ago and the genomes have been published. In contrast, the focus of diArk is to provide access to already existing genome assembly data and large cDNA/EST databases. This should enable researchers interested in comparative genomics, phylogeny, any other topic requiring taxonomic sampling, and single gene studies to get immediate access to most of the eukaryotic data available worldwide.

### Future Developments

At the moment, it is not planed to include species data from the other two domains of life, the bacteria and the archaea, although diArk provides the framework for an easy expansion. Instead, we plan to extend diArk's current eukaryotic data content and its technical basis. From the user perspective it would be advantageous to obtain more information about the data availability and the usability of the various project web-sites. In addition, we intend to include some sequencing related data like assembly versions and coverage that will help the user to judge the different datasets. On the technical site, we plan to provide an undo function for any search as well as a general email alert for updated database content.

## Conclusion

**Figure 2:**
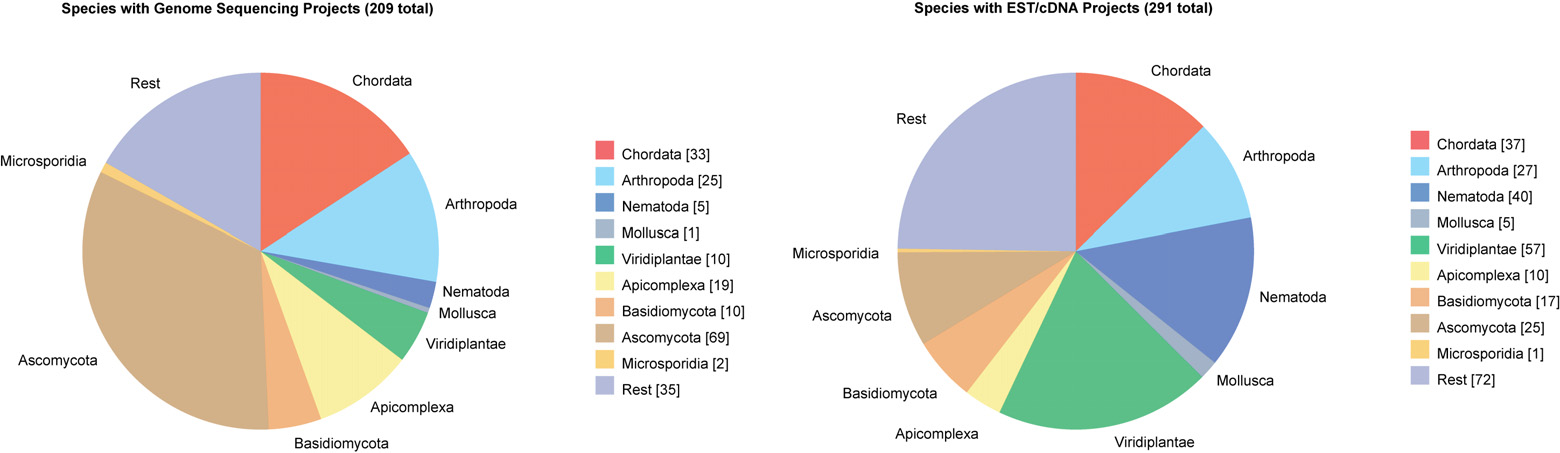
**Distribution of genome sequencing and cDNA/EST projects over major branches of eukaryotic life**. The numbers of sequencing projects for some major branches of eukaryotic life are shown. The charts show the bias towards certain branches originating from the various large-scale sequencing efforts. The total number of cDNA/EST and genome projects exceeds the number of species in diArk because for some species both data are available.

diArk is a new database to store, organize, and present the most relevant information about completed genome projects and EST/cDNA data from eukaryotes. The web-interface provides five search modules each with detailed options and three different views of the selected data. Currently, diArk provides information about 415 eukaryotic species, 823 sequencing projects, and 248 publications. cDNA/EST data is available for 291 species and genome assemblies have been released for 209 eukaryotes (13-Dec-2006; Figure 2). There are striking differences between the two diagrams: Due to large-scale efforts cDNA/EST data has been produced for many nematodes and plants while only a few of these species have been sequenced on a genomic basis. In contrast, the comparative genomic programs on fungi and protozoa pathogens have resulted in many complete fungi and apicomplexa genomes.

## Availability and Requirements

Project name: diArk – a resource for eukaryotic genome research

Project home page: 

Operating system: Platform independent

Programming language: Ruby

Other requirements: The current version of diArk requires Firefox version 1.5 or higher with cookies and JavaScript enabled. Currently, other browsers do not have the required feature set or do not comply with the standards of the W3C [11].

Web-service: To use the web service via SOAP, the WSDL-file can be obtained at . For using XML-RPC, users can connect to the endpoint URL .

Licence: The database schema, the web application and all scripts can be obtained upon request and used under a Creative Commons License.

Any restrictions to use by non-academics: Obtaining diArk by non-academics requires permission.

## List of Abbreviations

WSDL Web Services Description Language

SOAP Simple Open Access Protocol

XHTML Extensible HyperText Markup Language

XML Extensible Markup Language

XML-RPC XML-Remote Procedure Call

## Authors' contributions

MK specified the requirements from a user's perspective, defined the rules for data handling, and collected all the data. FO designed the database scheme and set up the technical requirements. FO and MH did the technical design and the programming. MK and FO wrote the manuscript. All authors read and approved the final manuscript.

## Supplementary Material

Additional file 1 **Database schema**. The file contains the detailed database schema. Click here for file
