# Supplementary figures and images for: diArk – a resource for eukaryotic genome research
Source: BMC Genomics. 2007 Apr 17;8:103. doi: 10.1186/1471-2164-8-103 (PMC1868023; doi:10.1186/1471-2164-8-103)

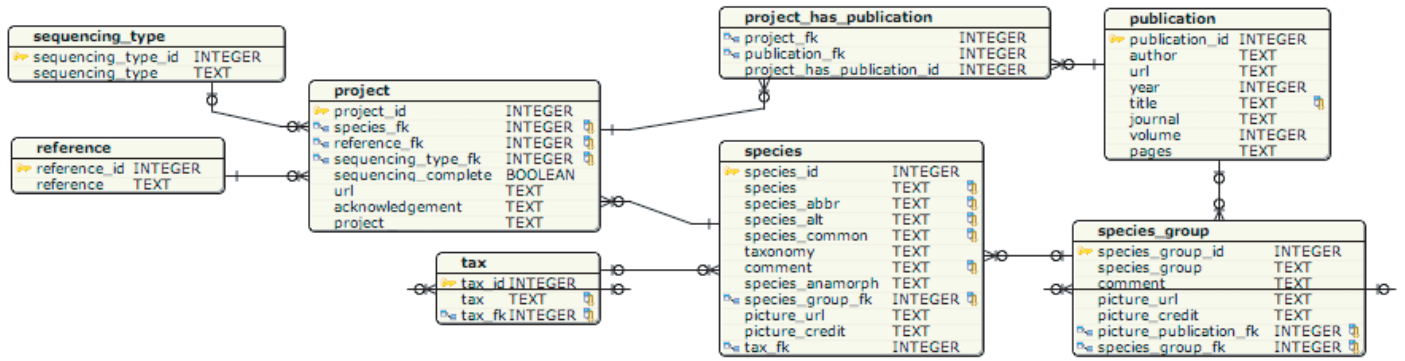

Supplement: Additional file 1 — Database schema. The file contains the detailed database schema. [file 1471-2164-8-103-S1.pdf]
